# Taxa-function robustness in microbial communities

**DOI:** 10.1186/s40168-018-0425-4

**Published:** 2018-03-02

**Authors:** Alexander Eng, Elhanan Borenstein

**Affiliations:** 1 Department of Genome Sciences, University of Washington, Seattle, WA 98102 USA; 2 Department of Computer Science and Engineering, University of Washington, Seattle, WA 98102 USA; 3 Santa Fe Institute, Santa Fe, NM 87501 USA

**Keywords:** Microbial community, Taxa-function relationship, Robustness

## Abstract

**Background:**

The species composition of a microbial community is rarely fixed and often experiences fluctuations of varying degrees and at varying frequencies. These perturbations to a community’s taxonomic profile naturally also alter the community’s functional profile–the aggregate set of genes encoded by community members–ultimately altering the community’s overall functional capacities. The magnitude of such functional changes and the specific shift that will occur in each function, however, are strongly dependent on how genes are distributed across community members’ genomes. This gene distribution, in turn, is determined by the taxonomic composition of the community and would markedly differ, for example, between communities composed of species with similar genomic content vs. communities composed of species whose genomes encode relatively distinct gene sets. Combined, these observations suggest that community functional robustness to taxonomic perturbations could vary widely across communities with different compositions, yet, to date, a systematic study of the inherent link between community composition and robustness is lacking.

**Results:**

In this study, we examined how a community’s taxonomic composition influences the robustness of that community’s functional profile to taxonomic perturbation (here termed *taxa-function robustness*) across a wide array of environments. Using a novel simulation-based computational model to quantify this taxa-function robustness in host-associated and non-host-associated communities, we find notable differences in robustness between communities inhabiting different body sites, including significantly higher robustness in gut communities compared to vaginal communities that cannot be attributed solely to differences in species richness. We additionally find between-site differences in the robustness of specific functions, some of which are potentially related to site-specific environmental conditions. These taxa-function robustness differences are most strongly associated with differences in overall functional redundancy, though other aspects of gene distribution also influence taxa-function robustness in certain body environments, and are sufficient to cluster communities by environment. Further analysis revealed a correspondence between our robustness estimates and taxonomic and functional shifts observed across human-associated communities.

**Conclusions:**

Our analysis approach revealed intriguing taxa-function robustness variation across environments and identified features of community and gene distribution that impact robustness. This approach could be further applied for estimating taxa-function robustness in novel communities and for informing the design of synthetic communities with specific robustness requirements.

**Electronic supplementary material:**

The online version of this article (10.1186/s40168-018-0425-4) contains supplementary material, which is available to authorized users.

## Background

The examination and characterization of microbial communities have become increasingly important as their impacts on human health, industrial processes, and the environment have been recognized. These communities have been studied both in terms of their taxonomic and functional compositions, elucidating important community features and revealing intriguing disease- and environment-associated variation. A community’s taxonomic composition is often determined via targeted 16S rRNA sequencing [1], a technique that uses hyper-variable regions of the 16S rRNA gene to identify the microbes present in a given community and estimate their relative abundances. Such taxonomic information can provide insight into inter-microbial or host-microbe interactions and facilitate the detection of shifts in community ecology that may be associated with host disease [2–6]. The functional composition of a community, in turn, can be estimated via whole metagenome shotgun sequencing followed by gene annotation. Using such data, gene-level analyses have provided insight into the functional capacities of various microbial communities [7, 8] and how those capacities change over time or vary with altered environmental conditions [9, 10].

**Fig. 1:**
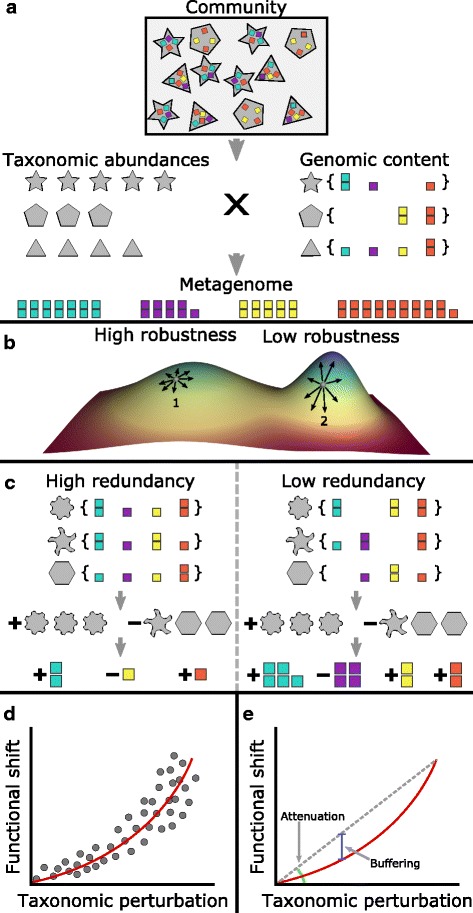
An illustration of the taxa-function relationship and response curves. **a** The functional profile of a community is a linear combination of the functional profile of each taxon in a community (the copy number of each gene in each taxon’s genome) weighted by the abundance of each taxon in that community. Note that here, a taxon need not be a species, but can instead represent any subpopulation of the community with shared genomic content (such as a single strain of a particular species). **b** The taxa-function relationship can be modeled as a high-dimensional landscape, linking each community composition to the corresponding functional profile. Here, we show an extremely simplified two-dimensional abstraction of this model to illustrate the impact of this landscape on taxa-function robustness. In this illustration, each coordinate on the plane corresponds to a specific taxonomic composition, with points close to one another corresponding to communities with similar taxonomic compositions. The height represents the functional profile of the community (and in this simplified illustration can denote, for example, the abundance of some function). Notably, the local topology of this landscape around a specific taxonomic composition (e.g., point 1) determines whether minor changes in that composition (represented as small movements on this plane; black arrows) will induce small (point 1) or large (point 2) functional shifts. **c** Depending on the exact distribution of genes across the genomes of species in a community, changes to the taxonomic composition of a community can produce functional shifts of varying magnitudes. For example, if the distribution of genes differs markedly between two communities (e.g., high vs. low redundancy), similar taxonomic composition perturbations to both communities may produce drastically different functional shifts. **d** To model the relationship between taxonomic perturbations and functional shifts in a given community, a taxa-function response curve is obtained by fitting a power function to a large array of measurements of functional shifts associated with many different taxonomic perturbations of varying magnitudes. **e** The taxa-function response curve can be decomposed into two factors: *attenuation*, which describes how quickly functional shifts increase in magnitude as taxonomic perturbations increase, and *buffering*, which indicates how well functional shifts are suppressed at smaller taxonomic perturbations

Indeed, these two facets of microbiome composition, namely its taxonomic structure and its functional capacities, offer different but complementary views into microbial communities. These two aspects of microbiome organization, however, are clearly not independent as the composition of genes in the metagenome is a direct derivative of the genes encoded by the community members’ genomes and the relative abundance of each member in the community. Moreover, this link can be represented as a simple set of linear equations wherein the abundance of each gene in the metagenome is the sum of that gene’s copy number in each community member’s genome weighted by the relative abundance of each community member (Fig. 1a) [11]. This inherent link between a community’s taxonomic composition and its functional profile, here referred to as the *taxa-function relationship* [12], has many practical applications in the analysis of microbial communities. For example, this relationship is explicitly utilized by tools such as PICRUSt [13] and Tax4Fun [14] for predicting overall community gene content based on the community taxonomic profile and available reference genomes. Other studies have similarly used the taxa-function relationship to identify taxonomic drivers of disease-associated functional shifts [15] or to estimate differences in community metabolic capacities [16]. Such methods for integrated analysis of multi-omic microbiome data offer unique, mechanistically driven insights into how taxonomic composition affects community function through features such as gene abundances and metabolism.

Conceptually, the taxa-function relationship can be viewed as a structure-to-function landscape, whose topology is determined by the distribution of genes across community genomes. As such, it is similar to the fitness landscape concept used in evolutionary biology [17–20], but instead of describing how changes in genotype map to changes in phenotype, it describes how changes in a microbial community’s composition map to changes in the community’s functional capacities. Characterizing the topology of this taxa-function landscape is similarly crucial for understanding how constraints on community ecology restrict community function and should be considered when designing the targeted manipulation of community composition. One important manifestation of the taxa-function landscape is the degree to which a shift in a community’s taxonomic composition will impact its functional capacities (a property that we refer to here as *taxa-function robustness*). Specifically, depending on the local topology of the taxa-function landscape around a given community, changes in the abundance of its members could result in a minor or major alteration to the community’s functional profile [21–24] (Fig. 1b; analogous to the impact of the fitness landscape on genetic robustness [25, 26]). As noted above, the topology of the landscape around a given community, and consequently, its taxa-function robustness depends solely on the manner in which genes are distributed among the genomes of that community’s members (Fig. 1c). For example, if a specific gene family (or pathway) is encoded by a single species in the community, any perturbation to that species’ abundance will directly translate to changes in the abundance of this gene family in the metagenome and ultimately in the community’s functional profile. If, however, this gene family is encoded by multiple species in the community, its abundance is less likely to substantially change in the face of taxonomic perturbations as a decrease in the abundance of one species that encodes this gene family could be compensated for by an increase in the abundance of another [21–24]. Moreover, if multiple genes tend to co-occur across the various genomes in a community [27], then those genes (and the functions associated with them) will shift in a similar manner as the taxonomic composition is perturbed. Combined, these observations suggest that the taxonomic composition of a community (which in turn also determines the distribution of genes across community members) directly impacts the taxa-function *robustness* of that community and may therefore vary substantially from community to community.

Importantly, this definition of taxa-function robustness aims to capture an important aspect of the taxa-function relationship and is independent of the specific variation the community actually experiences or its natural dynamics (just as a fitness landscape is determined by the genotype-to-phenotype relationship regardless of observed evolutionary trajectories). As such, it may not necessarily correspond to conventional notions of ecological robustness or to the resilience or stability of microbial community function. Yet, characterizing such underlying taxa-function robustness and its determinants is essential for gaining a profound understanding of community dynamics and function. For example, this feature of a community’s local taxa-function landscape could indicate how susceptible a community’s functional capacities are to stochastic fluctuations in the community’s composition and can be used as a null model when studying community dynamics in response to environmental change. More generally, while microbiome perturbations, be they minor stochastic fluctuations or major shifts in response to environmental modulation, are often characterized as ecological changes, in many cases, the most important consequences of these perturbations are shifts in overall community function. In the context of disease-associated dynamics, taxa-function robustness will also determine whether the functional capacities of a community could be maintained in the face of ecological dysbiosis. For example, the function of the gut microbiome is robust enough to maintain normal function despite day-to-day fluctuations in taxonomic composition [28], but may become disrupted following a more drastic perturbation as in the case of *Clostridium difficile* infection [29]. Determining a community’s functional robustness can further help estimate the functional impact of a planned targeted perturbation (e.g., via a particular probiotic) or evaluate candidate synthetic communities during the design process to gauge how susceptible they are to disruption of function.

Here, we set out to systematically characterize and study the taxa-function robustness of communities from diverse environments. This requires a comprehensive, systematic, and unbiased mapping of the local topology of the underlying taxa-function landscape around each community composition. Unfortunately, currently available experimental data does not adequately or comprehensively survey functional shifts associated with small changes to a particular taxonomic composition. We address this challenge by using a simulation-based approach, uniformly and systematically simulating a range of possible perturbations (including small perturbations) to each community’s taxonomic composition. This approach, combined with the prediction of community functional profiles associated with each such perturbation, allows us to generate a large set of perturbed compositions relative to each original community and to sample the taxa-function landscape around these communities. Given this simulation-based approach, below, we first define two factors that characterize a community’s taxa-function robustness. We then present an analysis of how taxa-function robustness varies within and between body sites in human-associated communities as well as across several non-host-associated communities. We extend this analysis to the robustness of individual functions and pathways, identifying universally robust functions and noting that environment-specific pressures may influence robustness variation in specific functions. Next, we investigate how the manner in which genes are distributed across microbial genomes is associated with a community’s taxa-function robustness and use this information to predict robustness directly from taxonomic composition. Finally, we confirm that our robustness estimates are in agreement with observed taxonomic and functional shifts measured from experimental data.

## Results

### Characterizing and defining taxa-function robustness

Consider the taxa-function mapping discussed above, linking a community’s taxonomic and functional compositions. To rigorously characterize how this mapping impacts taxa-function robustness, we define the *taxa-function response curve*, describing the average shift in the functional profile of a community as a function of the taxonomic perturbation magnitude and the stability of a community’s functional profile when faced with taxonomic perturbations (Fig. 1d). Response curves are commonly used in biology to describe how the changes in an organism vary as the magnitude of a particular stimulus is modulated (e.g., drug dosage-dependent physiological effects) and allow quantitative comparisons between the response curves of different individuals [30–33]. Interpreting a community’s taxa-function robustness via these response curves could offer insights such as the potential impact of antibiotics on a community’s functional capacities or the expected stability of candidate synthetic communities. For example, a community’s taxa-function response curve could be used to identify an antibiotics dosage threshold above which there would be significant disruption to community function. The specific form of the taxa-function response curve was chosen by comparing the fit of various functions to the relationship between taxonomic perturbation and functional shift magnitudes across all communities examined (Additional file 1: Supplementary Text; Additional file 2: Figure S1; Methods).

To provide a direct, quantitative, and interpretable comparison of taxa-function robustness differences between communities, we will further specifically focus on two robustness factors that can be derived from the response curve (Fig. 1e): The first factor is *attenuation* or how rapidly functional shift increases as perturbation magnitude increases. Attenuation describes the slope of the response curve and thus models the intuitive expectation that larger perturbations should generate larger functional shifts. Technically, attenuation is defined as inversely proportional to the response curve slope, implying that increased attenuation leads to smaller functional shifts and thus higher robustness. The second factor is *buffering* or how well functional shifts are suppressed at smaller perturbation magnitudes. Buffering determines how large a taxonomic perturbation must be before noticeable functional shifts occur. Higher buffering thus indicates that relatively large perturbations are required before a substantial functional shift could be observed. This factor is especially important when considering community robustness in the absence of major external changes, as buffering will determine whether small fluctuations in composition due to stochastic variation, neutral community dynamics, or minor environmental variation will significantly impact the community’s function. Indeed, many biological systems can completely buffer small perturbations, but may be more susceptible when exposed to larger perturbations [34, 35]. The precise definitions of the response curve and the two robustness factors are further elaborated in the “Methods” section.

### Taxa-function robustness varies within and between environments

To comprehensively characterize taxa-function robustness across the human microbiome and certain non-host-associated ecosystems, we obtained taxonomic compositions from previously assayed communities representing several distinct environments. Specifically, we considered four sites from the human microbiome, including 128 gut communities, 1141 oral communities (pooled from 9 subsites), 285 skin communities (pooled from 3 subsites), and 209 vaginal communities (pooled from 3 subsites), all obtained as part of the Human Microbiome Project [36]. Additionally, to extend our study beyond host-associated communities, we included 132 aquatic communities (pooled from 3 subsites) and 199 soil communities (from 4 subsites), collected in association with the Earth Microbiome Project [37]. For each community, we generated 4500 perturbed taxonomic compositions at varying magnitudes (using the weighted UniFrac distance between the original and perturbed community to measure the magnitude of perturbation). Compositional perturbations were simulated by randomly modifying the abundances of individual taxa in the original community such that the expected magnitude of change in each taxon’s abundance was proportional to its original abundance. We further filtered the obtained perturbations to uniformly sample perturbation at a range of taxonomic distances, resulting in an average of 933 ± 186 perturbations per community and a total of 1,954,447 perturbations (see “Methods” for complete details). To determine the functional profiles of both the original and perturbed compositions, we used PICRUSt [13], a computational framework for inferring the functional profile of a given community based on its taxonomic composition as described above. Using these inferred functional profiles, we measured the functional shift associated with each taxonomic perturbation and obtained a taxa-function response curve for each community as described in the previous section. We further calculated the associated attenuation and buffering values based on the response curve of each community (“Methods”). With this approach, we were able to compare response curves and robustness factors between communities and examine how taxa-function robustness varied within and between environments.

**Fig. 2:**
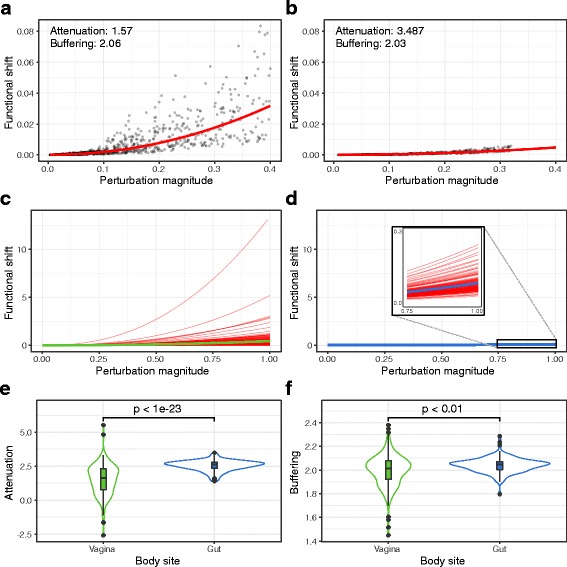
Examples of taxonomic perturbations, their corresponding functional shifts, and the associated response curves. The taxonomic perturbation and functional shift magnitudes generated for a single vaginal (**a**) and a single gut (**b**) community. Each point represents a single perturbation. The red lines indicate the taxa-function response curve fit to these points. The response curves for all vaginal (**c**) and gut (**d**) communities are overlaid to compare general body site trends. Green and blue lines represent the mean response curve for all vaginal and gut communities respectively. The robustness factor distributions associated with these response curves are shown as violin plots with inlaid boxplots for attenuation (**e**) and buffering (**f**). The width of the violin plot indicates the density of robustness factor values, the middle of the box displays the median robustness factor value, the upper and lower edges of the box represent the 75th and 25th percentiles, respectively, and the whiskers extend to 1.5 times the inner quartile range (range between the 75th and 25th percentiles) past either end of the box. Outliers are shown as individual black circles

To gain an intuition of how perturbation magnitudes affect the degree of functional shift, we first examined the taxa-function response curve of a single human vaginal community. As expected, the degree of the functional shift generally increased with the magnitude of the taxonomic perturbation (Fig. 2a). Notably, we observed some variation in the extent of functional shifts associated with taxonomic perturbations of a similar magnitude. Interestingly, comparing this response curve to the response curve for a community from a different environment–the gut–revealed marked differences, with the gut community displaying noticeably smaller functional shifts at similar taxonomic perturbation magnitudes (Fig. 2b). These differences are also apparent in the corresponding robustness factors, with the vaginal community having lower attenuation (1.57 compared to 3.487 in the gut) and comparable buffering (2.06 compared to 2.03). Moreover, the vaginal community also displayed more drastic deviations from its taxa-robustness response curve.

To examine whether the differences in robustness between vaginal and gut communities extended beyond these two specific examples, we compared the taxa-function response curves for all communities from these two body sites. This confirmed that vaginal communities indeed exhibited larger functional shifts on average compared to gut communities for similar taxonomic perturbation magnitudes (Fig. 2c, d). The gut communities also appeared to have more similar response curves across communities, whereas vaginal community response curves were more diverse. Examining the robustness factors of these communities further revealed a clear difference between these two body sites, with gut communities having significantly higher attenuation compared to vaginal communities (Fig. 2e; *p* < 10^−23^; Wilcoxon rank-sum test). Interestingly, however, we found only slightly higher buffering values in gut communities (Fig. 2f; *p* < 0.01; Wilcoxon rank-sum test). We further examined whether this marked difference in robustness between the vaginal and gut microbiomes can be attributed solely to the substantially lower diversity of vaginal microbiomes. To this end, we subsampled all communities from the vagina and gut to obtain communities with comparable diversity (“Methods”). We found that, in these subsampled communities, attenuation was still significantly higher in the gut compare to the vagina, which suggested that the difference in robustness could be attributed, at least partly, to some environment-specific features that go beyond community diversity (Additional file 3: Figure S2; *p* < 10^−6^; Wilcoxon rank-sum test).

**Fig. 3:**
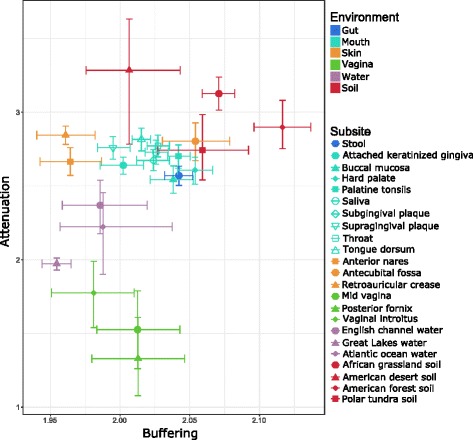
Comparison of attenuation and buffering between environments and subsites. Points represent the median attenuation and buffering values of samples from the indicated subsite, with error bars indicating the 95% confidence interval of the median in both dimensions (see “Methods”). Colors are shared by subsites from the same environment, while shape indicates the specific subsite within the environment

To extend this analysis beyond vaginal and gut communities, we next examined the robustness factors of every community from all environments we analyzed. For this analysis, we also separated HMP communities by subsite to determine how between-subsite robustness differences compared to differences between more distantly related environments. Our analysis revealed substantial variation in attenuation between environments, potentially suggesting different pressures to maintain robust functional profiles under different environmental conditions (Fig. 3). Notably, communities from all three vaginal subsites appear to have the lowest attenuation, with communities from other body sites exhibiting higher (and more comparable) attenuation. In contrast, communities from two of the three skin subsites appear to be among the most robust body site communities. Soil communities tend to have the highest attenuation, whereas marine communities have intermediate attenuation values. Interestingly, subsites from the same environment tend to cluster by attenuation and buffering, suggesting that spatially distinct subsites (such as different locations in the mouth) still exhibit similar taxa-function robustness factors, potentially reflecting shared environment-specific conditions. Buffering similarly exhibited some variation between environments but not as extreme as the variation seen in attenuation values; in the analyses below, we therefore focus mainly on attenuation to examine differences in robustness.

### Function-specific robustness reflects environmental conditions

Given the variation in overall taxa-function robustness observed above, we next set out to examine whether robustness also varied across different functions and whether such *function-specific robustness* is consistent across environments. To this end, we calculated function-specific response curves that described the average magnitude of the relative shift in a particular function’s abundance due to a taxonomic perturbation (see “Methods”). The resulting function-specific response curves can be analyzed as described above to obtain function-specific attenuation measurements.

**Fig. 4:**
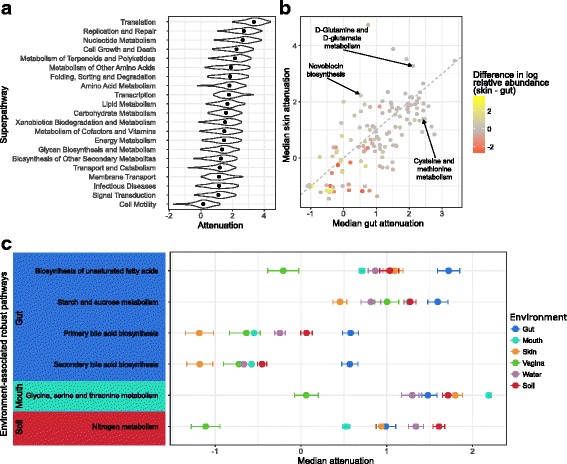
Attenuation of individual functions. **a** The density of attenuation values for each KEGG superpathway across all communities, with dots indicating the median attenuation. **b** A scatterplot of the median attenuation of each function in skin communities vs. gut communities. The color of each point indicates the difference in log median relative abundance of that function between skin and gut communities. The gray line indicates the 1:1 relationship in median attenuation. Only differentially abundant functions are show (FDR < 0.01; Wilcoxon rank-sum test). **c** Each point shows a function’s median attenuation in a particular environment with error bars displaying the 95% confidence interval and color indicating the environment

Examining function-specific attenuation at the superpathway level, we found marked variation in the robustness of different functions. Perhaps not surprisingly, superpathways associated with universal housekeeping activities, such as translation, nucleotide metabolism, and cell growth, were among the most robust functions, likely reflecting high redundancy in these functions across genomes in all communities and environments (Fig. 4a). In contrast, functions associated with a more specialized lifestyle, such as cell motility, transport, secondary metabolite biosynthesis, and glycan metabolism were generally less robust.

To further characterize differences in function-specific robustness across environments, we next compared the attenuation of each function between environments, this time analyzing functions at the pathway level. This analysis revealed similarly intriguing between-environment differences. Notably, the difference observed in a function’s robustness between environments often does not seem to be associated with the difference in function abundance between environments (Fig. 4b, Additional file 4: Figure S3). This finding suggests that the abundance and robustness of a given function may be driven by different pressures. Put differently, some functions may be beneficial at high capacity but can tolerate variation in their abundance, hence exhibiting high abundance but low robustness. In contrast, other functions may be advantageous at a more stable capacity, even though they are required at a relatively low capacity, and will hence exhibit high robustness but low abundance. For example, while cysteine and methionine metabolism occurs at low abundance in gut communities (1.5% median relative abundance; Additional file 5: Table S1), it is one of the most robust functions specifically in the gut (2.43 median attenuation; 7th most robust gut function; Fig. 4b, Additional file 6: Table S2). Indeed, cysteine and methionine deficiencies are associated with malnutrition and can be influenced in part by the gut microbiome [38], and accordingly, maintaining a stable capacity of genes from this pathway in the gut microbiome may be beneficial. Similarly, novobiocin biosynthesis had high robustness specifically in skin communities (2.23 median attenuation; 10th most robust skin function) and its high robustness could be related to novobiocin’s antibiotic activity against *Staphylococcus epidermidis* [39]. D-glutamine and D-glutamate metabolism also had high robustness in skin communities (3.28 median attenuation; 5th most robust skin function) while being very low in abundance (0.3% median relative abundance). Enrichment for D-glutamine and D-glutamate metabolism in skin communities has been linked to individuals prone to atopic dermatitis [40]. This may suggest that rather than having high robustness to maintain baseline capacity, D-glutamine and D-glutamate metabolism could have high robustness to prevent dramatic increases in abundance.

We finally investigated which functions displayed noticeably higher robustness in one or more environments compared to the others (see “Methods”). This analysis revealed many pathways that had significantly higher robustness in specific environments (Additional file 7: Figure S4), including biosynthesis of unsaturated fatty acids, primary and secondary bile acid biosynthesis, and starch and sucrose metabolism, which were all more robust in the gut than any other environment (Fig. 4c) and potentially reflected the increased occurrence of metabolites related to these functions in the gut compared to the other environments. In contrast, glycine, serine, and threonine metabolism was most robust in oral communities and could be related to the role of these amino acids in pH recovery in the oral environment after microbial fermentation of carbohydrates [41]. Interestingly, nitrogen metabolism had higher robustness in soil communities compared to the various body site communities and may reflect the role that these communities play in the nitrogen cycle.

### Gene distribution impacts taxa-function robustness across body sites

As discussed above, the observed variation in taxa-function robustness between and within environments likely reflects differences in the way various genes/functions are distributed across community members in each environment. To characterize how the distribution of functions contributes to robustness variation, we formulated a set of gene distribution features (GDFs), including *average functional redundancy*, *average functional similarity*, *average genome size*, *genome size variability*, and *unique function abundance*, to describe particular aspects of this distribution (see “Methods”). *Functional redundancy*, defined here as the redundancy of each function weighted by the relative abundance of that function, has been proposed as an important contributor to the robustness of a community’s functional capacities [21–24]. *Functional similarity*, defined here as the pairwise similarity in genomic content between species, captures the interchangeability of microbes and the potential for a change in the abundance of one species to be compensated for by an opposite change in the abundance of another. *Genome size*, measured here as the number of genes in a genome, accounts for the possibility that abundance changes in species with larger genomes will produce larger functional shifts. *Genome size variability* measured the presence of microbes with significantly different genome sizes, which could potentially decrease robustness due to the inability for such microbes to compensate for abundance changes in one another. Finally, *unique function abundance* measures the total abundance of functions that are encoded by a single species, aiming to capture the prevalence of functions with no redundancy (and hence with no potential for compensatory changes). Notably, while some of these GDFs are correlated with each other, these correlation magnitudes are < 0.5, suggesting that they indeed capture different aspect of functional distribution (Additional file 8: Figure S5). A precise mathematical definition of these features can be found in the “Methods” section. Importantly, though previous work has found some association between robustness and species diversity [42, 43], here, we wish to focus on the impact of functional distribution and hence exclude species diversity as a feature in this analysis.

**Fig. 5:**
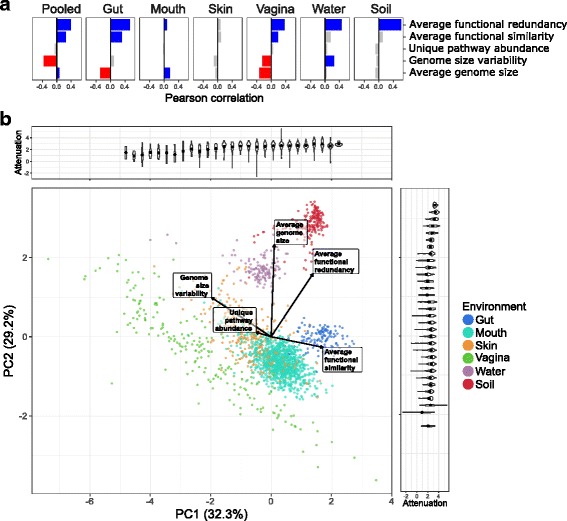
Associations between gene distribution features (GDFs) and taxa-function robustness. **a** Pearson correlations between GDFs and attenuation; blue and red bars indicate significant (*p* < 0.01) positive and negative correlation coefficients, respectively, whereas gray bars show non-significant correlation coefficients. Each panel corresponds to correlations when considering communities from all environments (pooled) or the subset of communities from a particular environment. **b** Communities plotted by the first two principal components (PCs) determined by principle component analysis (PCA) of the five GDFs. The percent variance explained by each PC is indicated on the axis labels. Loadings for GDFs are indicated by the direction and magnitude of the labeled vectors. Communities were binned along both axes, and the density of attenuation values is displayed by the width of the violin plots in the plots along the top and right margins of the PCA plot. Dots indicate the median attenuation of communities in each bin

We calculated these GDFs for each community and examined how they correlate with robustness both within and across environments, aiming to identify universal or environment-specific relationships with robustness. As expected, we found that functional redundancy positively correlates with attenuation both when communities from all environments are pooled together (*r* = 0.38; *p* < 10^− 72^) and within each environment individually (Fig. 5a). Additionally, functional similarity among community members appears to be positively associated with attenuation when environments are pooled together (*r* = 0.24; *p* < 10^− 29^), as well as in several individual environments (e.g., the gut). Genome size variability, which we hypothesized may negatively impact robustness, does exhibit a negative association with robustness when pooling communities from all environments together, and both genome size and genome size variability also have negative associations in specific environments (i.e., the gut or vagina). Interestingly, however, these associations are not consistent across all environments and may indicate that the relationship between these GDFs and robustness are in part influenced by other features of the community.

Obviously, the impact of each of the GDFs described above on robustness is not independent of the impact of other GDFs, and thus, their individual associations with robustness may not reflect how the combination of all five GDFs contributes to robustness. To examine how variation in attenuation is associated with the major axes of variation separating communities based on all GDFs simultaneously, we performed a principal component analysis (PCA) of the five GDFs described above (Fig. 5b). Remarkably, we found that the first two principal components of these GDFs clearly separate communities by environment, even though they are based on only five simple summary metrics of gene distribution without directly accounting for the presence or absence of specific microbes or functions. This finding indicates a strong environment-specific signature of gene distribution. We further found a significant positive correlation between attenuation and both the first and second principal components (Fig. 5b; *r* = 0.44; *p* < 10^−99^ and *r* = 0.09; *p* < 10^−4^ respectively), confirming that the variation in the combination of these GDFs is inherently associated with variation in community robustness. Given these findings, we additionally examined whether GDFs can be used to predict the robustness of each community, using both the set of 5 GDFs above, as well as an expanded set of 45 GDFs (Additional file 9: Table S3A-B). This analysis again demonstrated the strong association between gene distribution profiles and taxa-function robustness (though predictive power varied markedly between environments) and highlighted the importance of functional redundancy in determining robustness (Additional file 1: Supplementary Text, Additional file 10: Figure S6; Additional file 11: Figure S7, Additional file 12: Table S4).

### Taxa-function robustness estimations are in agreement with observed functional shifts

The above results rely on simulated taxonomic perturbations and predicted functional profiles (via PICRUSt) rather than on observed taxonomic shifts and shotgun metagenomic-based functional profiling. Clearly, there are caveats involved in both simulated perturbations (which may not reliably capture natural community fluctuations) and prediction accuracy. Yet, as described in the “Background” section, this simulation-based approach is crucial for comprehensively mapping the local taxa-function landscape. Specifically, this approach allows us to survey a large set of perturbed community compositions uniformly distributed around each community and sampled across a range of weighted UniFrac dissimilarities (ranging from 0.001 to 0.4)––a challenging task using available metagenomic data. Nonetheless, to confirm that our simulation-based estimates of robustness are biologically meaningful, here, we further examine whether they agree with observed shifts in communities’ taxonomic and functional profiles. To this end, we examined functional profiles that are based directly on shotgun metagenomic sequencing for a subset of the HMP communities used in our above analyses. Specifically, considering the HMP communities for which both a shotgun metagenome was available and the 16S rRNA data passed our quality filtering process (see “Methods” section), we were able to obtain 94 HMP communities from 5 different subsites with both taxonomic and metagenome-based functional profiles. Below, we use these communities to assess the agreement between predicted and metagenome-based functional profiles and between simulation-based robustness estimates and measured functional shifts. For the functional shift comparisons described below, communities from the same subsite were paired (resulting in 47 pairs), providing independent observations of functional shifts within each environment. Additionally, we were able to consider longitudinal shifts for a small number of communities (including 8 communities with 16S rRNA and metagenomic profiles sampled at two time points).

Using these data, we first verified that the compositions of the predicted functional profiles used in our analysis recapitulate metagenome-based functional profiles. To this end, we compared the dissimilarity between each predicted functional profile and its corresponding metagenome-based functional profile to the dissimilarity between each predicted functional profile and a different, randomly chosen metagenome-based functional profile from the same subsite. We found that indeed the median cosine dissimilarity between the corresponding predicted and metagenome-based functional profiles (at the pathway level) was significantly lower compared to the dissimilarity between predicted functional profiles and other metagenome-based profiles from the same subsite (*p* = 0.001, Wilcoxon rank-sum test). A Procrustes analysis [44] of the first two principal components of the functional profiles further demonstrated a significant fit between predicted and metagenome-based profiles (Procrustes measure of fit 0.89; *p* = 0.00001).

**Fig. 6:**
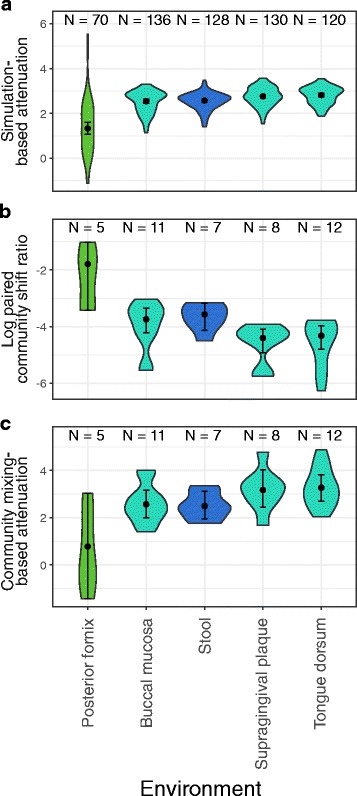
Robustness and metagenome-based functional shift trends across subsites. **a** Distribution of simulation-based attenuation estimates across five subsites, ordered by median attenuation in each subsite. **b** Distribution of the log scale ratio of metagenome-based functional profile dissimilarity to taxonomic profile dissimilarity observed between pairs of communities from the same subsite across five subsites. **c** Distribution of attenuation estimates from mixing community compositions between pairs of communities from the same subsite across five subsites

Next, we set out to examine the degree to which our simulation-based robustness estimates agree with observed functional shifts. In lieu of perturbed community compositions (and comparing the original and perturbed community compositions), we used pairs of communities (from the same subsite), considering one community as representing the original community and the other as representing a perturbed version of that original community. We then measured (for each pair of communities) the ratio between the dissimilarity in their functional profiles and the dissimilarity in their taxonomic profiles. This ratio is expected to be lower for more robust communities since taxonomic perturbations of similar magnitude should produce relatively smaller functional shifts in communities with higher taxa-function robustness. The findings of this analysis confirm our expectation above, demonstrating a noticeably lower robustness in vaginal communities (larger functional shifts relative to taxonomic perturbation magnitudes) compared to gut and oral communities (Fig. 6a, b). This suggests that the differences in attenuation values estimated from simulated perturbations reflect an inherent difference in how taxonomic changes induce functional shifts in communities from different environments.

Notably, as also discussed above, the taxonomic dissimilarities between different communities are substantially higher than the range of local taxonomic perturbations that are the focus of our study. Given this, the analysis above, using pairs of communities to represent original and perturbed communities may fail to capture more subtle properties of the local landscape of functional shifts around a community. To address this potential shortcoming and examine the impact of small taxonomic differences that are still rooted in metagenome-based functional profiles, we used a community-mixing approach. Specifically, given a pair of communities, a perturbed community composition at a specific (and small) taxonomic distance from the first community can be generated by identifying a mixing fraction and “replacing” this fraction of the first community’s taxonomic and functional profiles with a corresponding fraction of the second community’s taxonomic and functional profiles, respectively. This community-mixing approach allowed us to generate a set of perturbed community compositions at varying weighted UniFrac dissimilarities with metagenome-based functional profiles. We used this set of multiple perturbed compositions for each community to estimate the robustness of the first community using the same approach described in Fig. 1e. We found that these community-mixing metagenome-based attenuation values recapitulated the robustness trends identified using simulation-based attenuation estimates, with vaginal communities exhibiting significantly lower robustness than gut or oral communities, further supporting the agreement between simulation-based and metagenome-based robustness estimates (Fig. 6c).

Finally, we examined whether our robustness estimates agree with temporal changes in community composition. To this end, we used 8 HMP communities with taxonomic and metagenomic data available at two time points to measure longitudinal taxonomic and functional shifts (notably, relatively few HMP communities have both 16S rRNA and metagenome data available at two time points). As above, given a pair of communities from the same subsite from an individual obtained at two time points, we use the dissimilarity ratio between their functional and taxonomic profiles as a measure of observed taxa-function robustness, with the expectation that this ratio will decrease as robustness increases. Indeed, we found that attenuation was negatively correlated with this ratio (*r* = − 0.75). Furthermore, for each individual, we used the simulation-based robustness curve obtained for the community composition at the first time point, the metagenome-based functional profile of that community, and the observed taxonomic dissimilarly between the two time points to predict the expected metagenome-based functional shift between the two time points. Reassuringly, our expected functional shifts were positively correlated with the observed functional shifts (*r* = 0.86, *p* = 0.006), suggesting that these robustness curves are predictive of the relationship between the taxonomic perturbations and functional shifts that might be expected to occur over time in microbial communities.

## Discussion

Microbial communities are recognized as important components of various systems including human health, environmental resource cycling, and industrial processes. Given these varied and significant roles, improving our understanding of these systems requires a better comprehension of how these communities are structured taxonomically, how they function, how they react to change, and the relationships between these features. Previous work using the inherent link between taxonomic structure and function in microbial communities has already led to intriguing results and powerful tools for analyzing microbiome data [13–16]. However, a crucial property that has not yet been comprehensively studied is how a community’s underlying taxonomic structure modulates functional robustness in response to taxonomic perturbation. This taxa-function robustness is a direct derivative of community composition and of the distribution of genes across genomes, and while it may not correspond directly to conventional concepts of microbial community resilience and stability, it allows us to quantify an inherent component of the structure-function relationship between taxonomic and functional profiles.

Our analysis of robustness across various environments revealed intriguing differences between communities from different body sites. One of the more marked differences was between gut and vaginal communities, and while robustness to taxonomic perturbation has not been directly compared between gut and vaginal communities experimentally, there may be some evidence that supports vaginal communities being more susceptible to disrupted function than gut communities [16, 45]. We also observed that skin communities from two subsites were among the most robust host-associated communities. This functional robustness to taxonomic perturbation is especially intriguing given recent observations concerning the taxonomic stability of the skin microbiome despite virtually continuous perturbation [46]. Indeed, the link between ecological stability and taxa-function robustness is likely complex and multi-faceted since on the one hand high taxonomic stability may render taxa-function robustness unnecessary for maintaining community function, but on the other high taxa-function robustness may promote taxonomic stability via maintenance of the community niche.

We further found that functional redundancy was strongly associated with taxa-function robustness, in agreement with previously suggested hypotheses regarding the role of functional redundancy in microbial communities [21–24]. Other GDFs also showed some associations with robustness, but these associations were inconsistent across environments and may point to between-GDF interactions in determining robustness that masks a more consistent association with robustness. Notably, however, the five GDFs we examined appear to separate environments along different principal components, suggesting that they capture key information about environment-specific differences in community structure. This is even more striking when considering that these GDFs do not directly mirror the presence or absence of certain taxa or functions. This GDF-based separation may therefore suggest that communities inhabiting different environments substantially differ in the way community members contribute to overall community functional capacities. For example, the higher functional redundancy and functional similarity in gut communities might indicate that many community members in the gut are performing similar functions with relatively few specialized roles, whereas the increased presence of pathways unique to specific microbes and lower function redundancy in vaginal communities could point to a more well-defined and distinct functional niche in this environment.

To some extent, the extreme variation observed above in taxa-function robustness across environments may not be surprising. Some of this variation, for example, can be attributed to stochastic aspects of community assembly, such as priority effects or the pool of species from which community founders could originate. Such stochastic factors could lead to marked variation in community composition, and consequently to variation in taxa-function robustness. However, the similarity in robustness within a similar environment or subsite compared to between-environment robustness differences suggests that some of this observed variation may be selected for and that selection for robustness varies across environments. Selective pressure for robustness could vary based on the relative needs for consistency versus plasticity in community function when communities respond to changing environmental conditions or based on how consistent the environment might be. Indeed, community-level functional plasticity could be driven by changes in the metabolism or behavior of individual microbes in response to environmental changes, but could also be achieved by shifts in community composition that modulate the community functional profile in a desired direction. Indeed, noticeable alterations to taxonomic composition often accompany large environmental changes, such as in the gut microbiota following diet switches [47], in skin communities during and after atopic dermatitis flares [48], or in the vaginal microbiome during pregnancy [49], and in certain cases these composition alterations have also been associated with changes in community functional capacities [50, 51]. In such cases, lower taxa-function robustness may be selected to enable plasticity in community function, thereby allowing community function to adapt to a changing environment.

Notably, there are a few caveats to our taxa-function robustness estimation approach. First, the simulation-based perturbation method we used was fairly simple and restricted perturbations to only modify the abundances of present microbes without considering the possible addition of novel microbes, e.g., via migration. In reality, the underlying taxa-function landscape depends not only on the taxa that are present in a community but also on the taxa that could be introduced to the community from an environmental reservoir or transferred from some other source. Yet, the correspondence between our robustness estimates and the observed taxonomic and functional shifts in real communities suggests that the local topology of the taxa-function landscape around a community may be relatively similar when considering the possible influx of new taxa. Beyond the simulation-based perturbation method, our robustness calculations also depend on community functional profile prediction via a database of annotated microbial genomes. These predicted functional profiles may not accurately reflect the true functional profile of the community or variation between communities. For example, our analyses above have considered community members at the species level, ignoring potential strain-level variation [52]. While our taxa-function mapping representation and our approach for robustness estimation can be applied, in principle, at higher taxonomic resolution (e.g., profiling communities at the strain level and associating each strain with a corresponding distinct genome) when such data is available, failing to do so may introduce inaccuracies to the predicted functional profiles and subsequently to our robustness estimates. More generally, robustness estimates can be inflated if rarer functions (that are likely less redundant) are left unannotated or deflated if certain functions are actually highly redundant but missing from some genome annotations. It is worth noting, however, that despite these caveats, our robustness estimates were shown to agree with differences in taxonomic and functional profiles between naturally occurring community compositions, both across communities and over time.

## Conclusions

Our analysis of simulated community perturbations indicates that taxa-function robustness of microbial communities varies by environment, though subsites from within a given environment tend to share similar taxa-function robustness signatures. Furthermore, function-specific robustness at the pathway level is associated with the universality of the pathway, with microbial housekeeping pathways displaying higher robustness than pathways associated with more specialized lifestyles. Interestingly, the variation in the robustness of a pathway across communities was not associated with differences in pathway abundance. We also found that environment-specific characteristics of gene distribution across community member genomes account for between-environment differences in taxa-function robustness and suggest potential drivers for functional robustness. Finally, a comparison between simulation-based robustness estimates and metagenome-based taxonomic and functional shifts suggested that our robustness estimates agree with observed community dynamics.

Importantly, the applications for computational robustness estimation could extend beyond the analyses and results presented here. Function-specific robustness estimation, for example, could inform the analysis of novel communities from a particular environment, highlighting functions whose capacities are more or less robust than expected. Taxa-function robustness estimation could also be incorporated into the construction of synthetic microbial communities to inform the design of more resilient community compositions. As we further explore and analyze the temporal dynamics of microbial community structure and function, being able to determine how and why robustness varies will continue to be of interest.

## Methods

### Samples and data processing

The samples were obtained from the Human Microbiome Project [36] and the Earth Microbiome Project [37]. 16S rRNA-based operational taxonomic unit (OTU) tables and metadata files were downloaded from the QIITA website (http://qiita.microbio.me), which provides OTU tables generated with the QIIME workflow [53] using Greengenes OTUs [54]. OTU tables were filtered to remove read counts mapped to plant chloroplasts. To improve quality and comparability between taxonomic profiles, taxonomic profiles with fewer than 10 OTUs or fewer than 5000 reads were removed and the remaining taxonomic profiles were rarefied to 5000 reads. When analyzing body subsites, left and right bilaterally symmetric body subsites (antecubital fossa or retroauricular crease) were pooled together. For each subsite, communities were selected from different hosts. The resulting number of communities after each step in the filtering process and the final set of communities can be found in Additional file 13: Table S5.

### Functional profile prediction

The Kyoto Encyclopedia of Genes and Genomes (KEGG) [55, 56] was used to define orthologous gene functions in terms of KEGG Orthology (KO) groups. KO abundances were predicted using PICRUSt [13] tables to first normalize each OTU’s relative abundance by its 16S rRNA copy number and then infer KO abundance from the genomic content of each OTU. KO abundances were then normalized using inter-sample MUSiCC. Pathway-level functional summaries, as defined by the KEGG BRITE hierarchy [57], were obtained by evenly distributing each KO’s average copy number across all pathways that contain that KO. Both KO and pathway tables were filtered to remove non-bacterial orthologs.

### Taxonomic composition perturbation

Community taxonomic composition perturbations were designed to simulate stochastic OTU relative abundance fluctuations (assuming no migration or introduction of OTUs absent in the original community). Perturbation size for each OTU was proportional to the abundance of that OTU. Formally, for a given community with *N* OTUs with non-zero relative abundances, *a*_*i*_ ∀ *i* ∈ 1, 2, …, *N*, perturbation multipliers *m*_*i*_ ∀ *i* ∈ 1, 2, …, *N* were sampled from a uniform distribution over the interval (0, *M*], *M* being the maximum perturbation magnitude, and perturbation directions *d*_*i*_ were chosen such that *d*_*i*_ ∈ {−1, 1} ∀ *i* ∈ 1, 2, …, *N* with an equal chance of either direction. Given these values, the perturbed taxonomic composition OTU abundances *p*_*i*_ ∀ *i* ∈ 1, 2, …, *N* were calculated as

$$ {p}_i={a}_i\times {m}_i^{d_i} $$

and then renormalized such that ${\sum}_i^N{p}_i=1$. Using this method, each community was perturbed at 45 maximum perturbation magnitudes, *M*, evenly spaced between 1.2 and 10 inclusive with 100 perturbations at each maximum perturbation magnitude.

### Perturbation magnitude calculations

Changes in taxonomic composition between an original community composition and a perturbed composition were measured using the weighted UniFrac metric [58], a common, phylogeny-aware metric for estimating dissimilarity between microbial community compositions. The shift in a community’s functional profile was defined as the cosine dissimilarity between the original and perturbed functional profiles, as done in [59]. Specifically, given an original community and perturbed community with *N* unique pathways with average copy number *a*_*j*_ ∀ *j* ∈ 1, 2, …, *N* and *b*_*j*_ ∀ *j* ∈ 1, 2, …, *N*, respectively, the cosine dissimilarity between the two functional profiles is

$$ 1-\frac{\sum_j^N{a}_j{b}_j}{\sqrt{\sum_j^N{a}_j^2}\sqrt{\sum_j^N{b}_j^2}}. $$

### Robustness metric definition and fitting

For this study, the taxa-function robustness of a microbial community is defined as the average shift in the functional profile given a perturbation to the community’s taxonomic composition. To allow quantitative robustness comparisons between communities, we assume that the relationship between taxonomic perturbation magnitude and functional profile shift behaves as

$$ f=\frac{1}{e^a}{t}^b $$

where *t* denotes the magnitude of the taxonomic perturbation, *f* denotes the expected shift in functional profile, and *a* and *b* are community-specific coefficients. We term this function the taxa-function response curve. We further term the coefficient *a* attenuation since it describes the expected rate at which increases in the taxonomic perturbation magnitude are expected to increase functional profile shifts. We similarly term the exponent *b* buffering since it indicates how large a perturbation must be before a functional profile shift becomes noticeable and approaches the expected shift magnitude defined by attenuation. Other models to describe the relationship were tested, but the model presented here provided the best explanation of the taxonomic and functional dissimilarity relationship across all communities (Additional file 1: Supplementary Text; Additional file 2: Figure S1; Additional file 14: Table S6). Function-specific robustness was defined in a similar manner to taxa-function robustness except that instead of the cosine dissimilarity between the original and perturbed functional profiles, the functional shift of a single function was measured as the relative change in the abundance of that function.

Attenuation and buffering were fit by first transforming weighted UniFrac and cosine dissimilarities to the natural log scale to reduce heteroscedasticity observed in the simulated perturbation data (variance of cosine dissimilarity increased as weighted UniFrac distance increased). A uniform sampling of simulated perturbations across weighted UniFrac dissimilarities was obtained by subsampling perturbations across 50 non-overlapping windows evenly spaced on the natural log scale between minimum and maximum distances. For each community, in each window, perturbations were subsampled to 50 when ≥ 50 perturbations were present and all perturbations were kept when < 50 were present. The transformation to the natural log scale also transformed the proposed taxa-function robustness curve function to the following form:

$$ \ln (f)=-a+b\ln (t) $$

which was then fit using the linear least-squares best fit to calculate attenuation and buffering.

Due to the asymmetric distributions of attenuation and buffering, the pseudomedian was used rather than the median and pseudomedian attenuation and buffering estimates were calculated using the Hodges-Lehmann statistic [60]. The 95% confidence interval for a pseudomedian estimate was calculated as the range of values for which the Wilcoxon statistic (given the observed attenuation or buffering values) was between the 0.025 and 0.975 quantiles of the standard normal distribution.

### Function-specific most robust environment determination

A function was determined to be most robust in a particular environment (or environments) by comparing the median attenuation of that function across all environments using Mood’s Median test. Specifically, a function was defined as most robust in a set of environments if, for each environment in that set, the median attenuation of the function in that environment was both significantly higher than its median attenuation in all of the environments not in the set and not significantly different than its median attenuation in any of the other environments in the set.

### OTU subsampling procedure

To determine the relationship between diversity and robustness when comparing vaginal and gut communities, we subsampled communities from each environment to obtain similar species richness and repeated our analysis of between-environment robustness differences. Specifically, each community was randomly subsampled to 10 OTUs such that the probability of an OTU remaining in the subsampled community was proportional to its relative abundance in the original community. This subsampling procedure aimed to achieve a similar distribution of relative abundance among community members between the original and subsampled communities. Once subsampled, OTU abundances were renormalized.

### Gene distribution feature (GDF) definitions

As noted above, the five main gene distribution features (GDFs) used in the correlation and PCA analysis were average functional redundancy, average functional similarity, average genome size, genome size variability, and unique function abundance. Each GDF captures a different, though potentially related, aspect of the distribution of genes across the genomes of species in a community.

The functional redundancy of a given function was defined here as the evenness (Shannon’s diversity index) of the abundances of each species that encodes that function weighted by the copy number of the function in each species’ genome respectively:

$$ -\sum \limits_{i=1}^N\left[\left({s}_i{c}_i\right)\ln \left({s}_i{c}_i\right)\right] $$

where *N* species encode a function, *s*_*i*_ is the abundance of species *i* that encodes that function, and *c*_*i*_ is the copy number of that function in species *i*’s genome. This definition aims to capture how evenly species contribute to a function’s abundance, such that a function should be considered less redundant if one species contributes the majority of that function’s abundance while it will be more redundant if many species all contribute similarly to its abundance. The average functional redundancy of a community was then defined as the average of all functions present in a community, weighted by the relative abundance of each function in the community’s functional profile.

The functional similarity between two microbes aimed to capture how well two different species could compensate functionally for one another, and thus was defined as the cosine similarity between the functional profiles of two species:

$$ \frac{\sum_{i=1}^M{a}_i{b}_i}{\sqrt{\sum_{i=1}^M{a}_i^2}\sqrt{\sum_{i=1}^M{b}_i^2}} $$

where *M* is the number of functions encoded by at least one of the species, *a*_*i*_ is the copy number of function *i* in species *a*, and *b*_*i*_ is the copy number of function *i* in species *b*. The average functional similarity within a community was then defined as the unweighted average of all pairwise functional similarities between species present in the community.

Genome size for a given species was defined as the total abundance of functions encoded by the species (i.e., the sum of the copy number of each function in that species genome). Average genome size was calculated as the unweighted average of each species’ genome size and genome size variability was calculated as the coefficient of variation of species genome size.

Unique function abundance was defined as the total abundance of functions in a community’s functional profile that are each encoded by a single species (though each unique function need not be encoded by the same species).

### Metagenome-based data and functional shifts

Shotgun metagenome-based KO profiles for 94 communities were obtained from HMP. KO profiles were corrected using inter-sample MUSiCC [61] and summarized to the pathway level using the same protocol used for predicted profiles. To obtain shotgun metagenome-based functional profile differences, 47 random community pairs were assigned from the 94 communities with both 16S rRNA and shotgun metagenome profiles. Pairs were restricted to contain two communities from the same subsite. Between-community taxonomic and functional dissimilarities were calculated (as described above) between the communities in each pair.

### Mixed community perturbations

Community mixing was performed using the same community pairs as for metagenome-based functional shift measurements. For each pair, one community was designated the original community and the other the mixing community. For a taxon *i* with abundance *a*_*i*_ in the original community and abundance *b*_*i*_ in the mixing community, taxon *i*’s abundance in the mixed community perturbation (relative to the original community) with mixing fraction *m* was [*a*_*i*_(1 − *m*)] + [*b*_*i*_*m*]. Similarly, for a function *j* with average copy number *c*_*j*_ in the original community and average copy number *d*_*j*_ in the mixing community, function *j*’s abundance in the mixed community perturbation (relative to the original community) with mixing fraction *m* was [*c*_*j*_(1 − *m*)] + [*d*_*j*_*m*]. Mixed community perturbations were generated at specific weighted UniFrac dissimilarities by using a binary search to identify the mixing fraction that achieved a mixed community perturbation with the desired weighted UniFrac dissimilarity from the original community within a tolerance of 10^−9^. To fit robustness curves for the original communities, mixed community perturbations were generated at weighted UniFrac distances of 0.01 to 0.1 in intervals of 0.01 for each community pair (except for 1 community pair, which could not achieve a weighted UniFrac distance of 0.1 through community mixing). Robustness curves were then fit to the resulting perturbation taxonomic and functional dissimilarities as described above.

### Longitudinal functional shift calculations

Longitudinal data with 16S rRNA and shotgun metagenomic profiles collected at two time points were obtained for 8 HMP communities. Taxonomic and functional dissimilarities between the community compositions at each time point were calculated as above. Expected functional shifts based on robustness estimates were calculated using the robustness curve formula defined above, the estimated attenuation and buffering values for the community, and the weighted UniFrac distance between the community compositions at the two time points.

## Additional files

Additional file 1: Supplementary text. Description of candidate taxa-function response curve models and predictive model results. (DOCX 17 kb)

Additional file 2: **Figure S1.** Candidate taxa-function response curve model fits. (PDF 206 kb)

Additional file 3: **Figure S2.** Taxonomic perturbations, their corresponding functional shifts, and the response curves fit in gut and vaginal subsampled communities. (PDF 1487 kb)

Additional file 4: **Figure S3.** Comparison of pathway-specific attenuation trends between environments. (PDF 1253 kb)

Additional file 5: **Table S1.** Median relative abundance of pathways by environment. (XLSX 26 kb)

Additional file 6: **Table S2.** Median attenuation of pathways by environment. (XLSX 25 kb)

Additional file 7: **Figure S4.** Pathway-specific attenuation of all pathways by environment. (PDF 1657 kb)

Additional file 8: **Figure S5.** Correlations between gene distribution features. (PDF 45 kb)

Additional file 9: **Table S3.** Extended definitions of variables used in gene distribution features and list of gene distribution feature names. (XLSX 48 kb)

Additional file 10: **Figure S6.** Performance of GDF-based predictive models of attenuation, using an extended set of 45 GDFs. (PDF 1694 kb)

Additional file 11: **Figure S7.** Performance of GDF-based predictive models of attenuation with only 5 GDFs. (PDF 1677 kb)

Additional file 12: **Table S4.** Importance of GDFs by predictive model, environment, and replicate. (XLSX 5833 kb)

Additional file 13: **Table S5.** Number/fraction of samples remaining after each quality filtering step. (XLSX 10 kb)

Additional file 14: **Table S6.** Candidate taxa-function response curve function definitions. (XLSX 16 kb)

## Abbreviations

EMP: Earth Microbiome Project
GDF: Gene distribution feature
HMP: Human Microbiome Project
KEGG: Kyoto Encyclopedia of Genes and Genomes
KO: KEGG Orthology group
OTU: Operational taxonomic unit
