# Supplementary material for: Taxa-function robustness in microbial communities
Source: Microbiome. 2018 Mar 2;6:45. doi: 10.1186/s40168-018-0425-4 (PMC5833107; doi:10.1186/s40168-018-0425-4)
Supplement: Supplementary file 1 — Supplementary text. Description of candidate taxa-function response curve models and predictive model results. (DOCX 17 kb) [file 40168_2018_425_MOESM1_ESM.docx]

# Additional file 1: Supplementary Text

## Taxa-function response curve model evaluation suggests a power function relationship

We considered eight different models describing the relationship between a taxonomic perturbation magnitude,$t$, and the associated degree of functional shift, $f$. These models were constructed by first considering the expected increase in functional shift as taxonomic perturbation magnitudes increase, which suggests a potential linear component and is represented by the $a$ parameter in each model. The second consideration for each model was whether there would be a polynomial or generally non-linear component to the relationship. For this second component, we tested: the absence of a second component (model 1), a quadratic relationship (model 2), a more general power function relationship (model 3), a cosine transformation to match the measure of functional shift (model 4), a combination of linear and quadratic relationships (model 5), an exponential relationship (model 6), a combination of linear and exponential relationships (model 7), and an exponential relationship with a proportionality constant (model 8), using the variable $b$ to parameterize this second component when necessary (**Additional file 14: Supplementary Table 6**).

To evaluate these models, for every community we fit a response curve using each model and calculated the error for every perturbation of that community. We then aggregated communities by environment, by subsite, and pooled together across environments and determined the root mean squared error (RMSE) of each aggregate. Across all aggregates, model 3 had the lowest RMSE and model 2 consistently had the second lowest RMSE (**Additional file 7: Supplementary Figure 4**). Given that model 3 was a generalization of model 2, we decided to use model 3 to examine whether the variation in $b$ was associated with any biological factors.

## Predictive models support the association between robustness and functional redundancy

In addition to correlation analysis and PCA, we further examined associations between GDFs and robustness using predictive models. Specifically, we attempted to predict a community’s attenuation using that community’s GDFs and determined GDF associations with robustness based on the importance of each GDF in the model (i.e. a GDF being more informative suggests a stronger association). In this analysis, we tested both the 5 GDFs used in the main results as well as an expanded set of 45 GDFs (**Additional file 9: Supplementary Table 3A-B**). The additional 40 GDFs aimed to capture other aspects of gene distribution such as how many species encode unique functions, how many species encode each function, and the similarity in function co-occurrence across genomes. We evaluated models by using 70% of the available communities for model fitting and then measuring the correlation between observed and predicted attenuation, as well as the root mean squared error in attenuation prediction, with the remaining 30%. We also accounted for potential variation in performance due to the specific communities in model fitting and evaluation sets by repeating this process for 500 different paired sets of model fitting and evaluation communities, taking the average of our evaluation metrics across all 500 models. To ensure comparability between GDFs, GDFs were centered and scaled before model construction.

We found that predictions were significantly improved when we used the expanded GDF set to fit unregularized linear models to the pooled set of communities (**Additional file 10: Supplementary Figure 6**; **Additional file 11: Supplementary Figure 7**; p < 10^-101^; F-test) and all following analyses are based on models fit using the expanded GDF set. Unregularized linear model predictions on evaluation communities were somewhat correlated with observed community attenuation (**Additional file 10: Supplementary Figure 6A**; *r* = 0.63; RMSE = 0.54), indicating that at least a subset of these 45 GDFs were associated with robustness. When we attempted to regularize the linear models, we found no noticeable improvement in performance (**Additional file 10: Supplementary Figure 6B**; *r* = 0.62; RMSE = 0.54). We suspected that the relationship between GDFs and robustness might not necessarily be linear, so we also attempted to fit another model, a Regularized Random Forest [53], using 5000 trees for model construction. The Regularized Random Forest did have better predictive power than the linear models (**Additional file 10: Supplementary Figure 6C**; *r* = 0.7; RMSE = 0.49), suggesting that some GDFs may contribute to robustness in a nonlinear fashion. We also considered the possibility that GDF associations with robustness might vary by environment, so we fit Regularized Random Forests for each environment individually. This revealed noticeable variation in model performance across environments, with the best correlation between observed and predicted attenuation in water-associated communities (**Additional file 10: Supplementary Figure 6D**; *r* = 0.78) and the lowest prediction error for gut communities (RMSE = 0.33).

To examine robustness associations with specific GDFs, we next examined the importance of each GDF in each model. For linear models, feature importance was measured as the absolute value of the feature’s coefficient in the model, while Regularized Random Forests feature importance was measured as the mean decrease in accuracy when the feature’s values are permuted. Functional redundancy appeared as the most important feature in the Regularized Random Forest, further supporting its strong association with robustness, while the most important features in the linear model were related to function co-occurrence across genomes (**Additional file 12: Supplementary Table 4**).
