# Supplementary figures and images for: Taxa-function robustness in microbial communities
Source: Microbiome. 2018 Mar 2;6:45. doi: 10.1186/s40168-018-0425-4 (PMC5833107; doi:10.1186/s40168-018-0425-4)

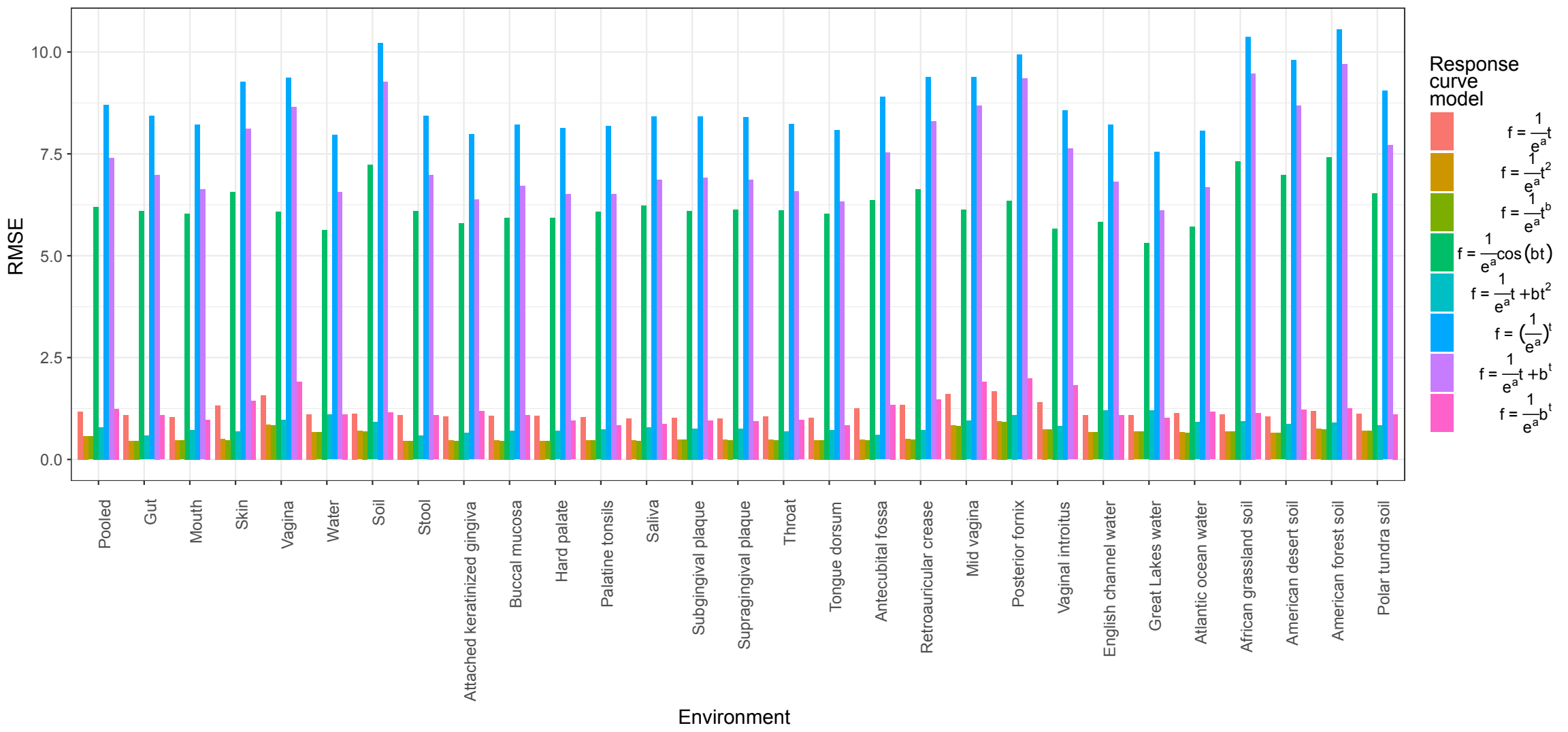

Supplement: Supplementary file 2 — Figure S1. Candidate taxa-function response curve model fits. (PDF 206 kb) [file 40168_2018_425_MOESM2_ESM.pdf]

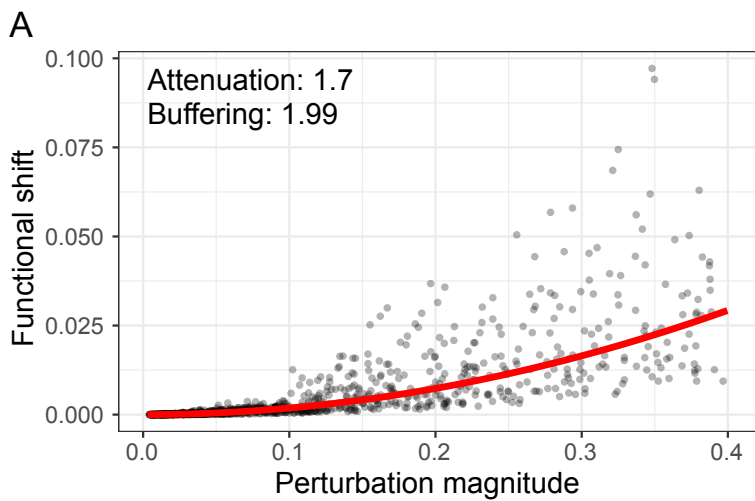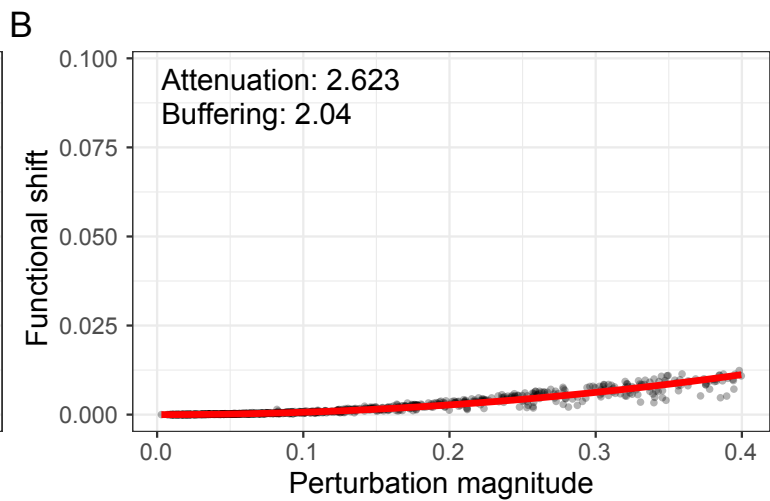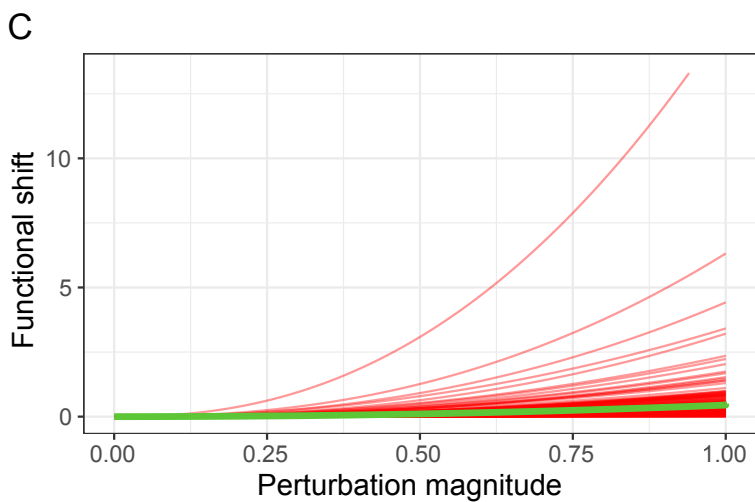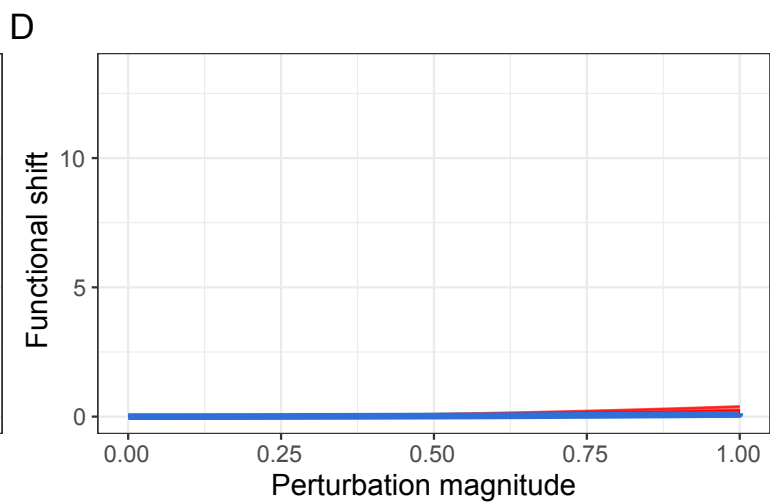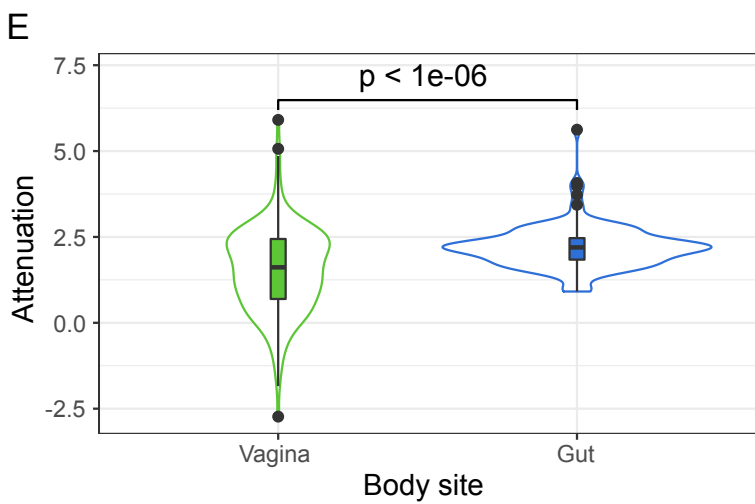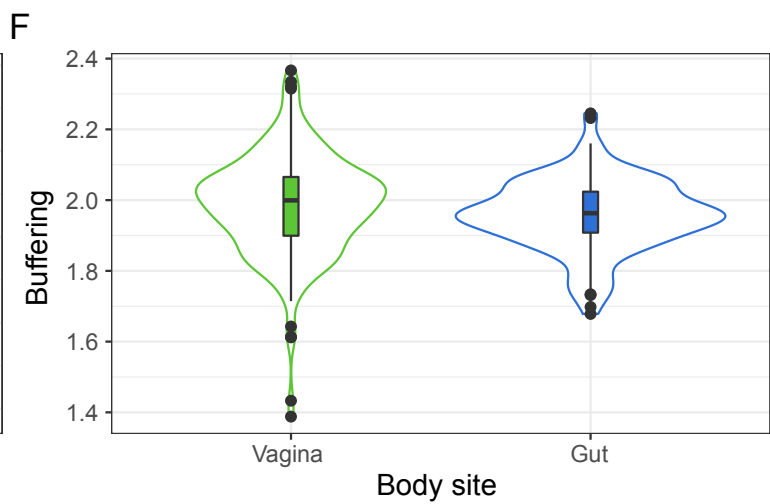

Supplement: Supplementary file 3 — Figure S2. Taxonomic perturbations, their corresponding functional shifts, and the response curves fit in gut and vaginal subsampled communities. (PDF 1487 kb) [file 40168_2018_425_MOESM3_ESM.pdf]

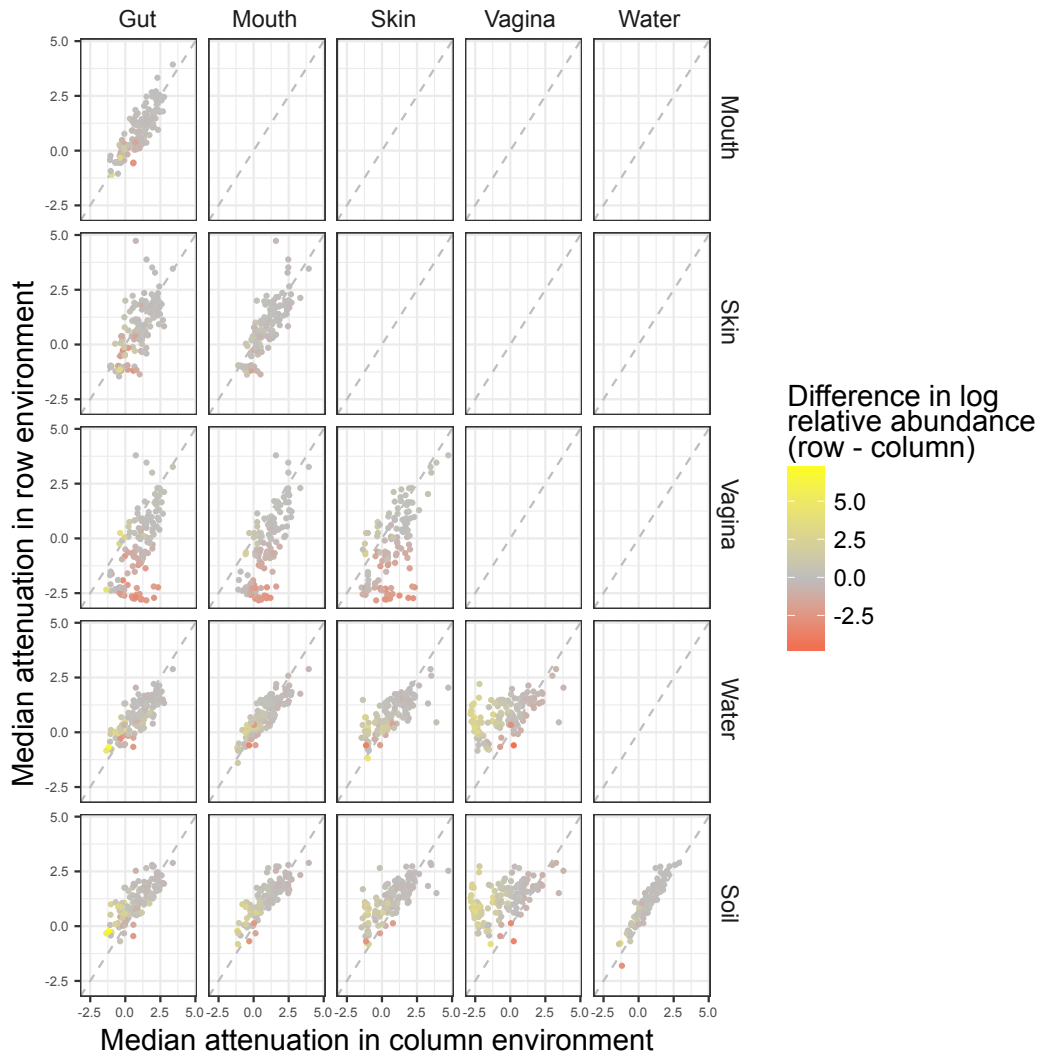

Supplement: Supplementary file 4 — Figure S3. Comparison of pathway-specific attenuation trends between environments. (PDF 1253 kb) [file 40168_2018_425_MOESM4_ESM.pdf]

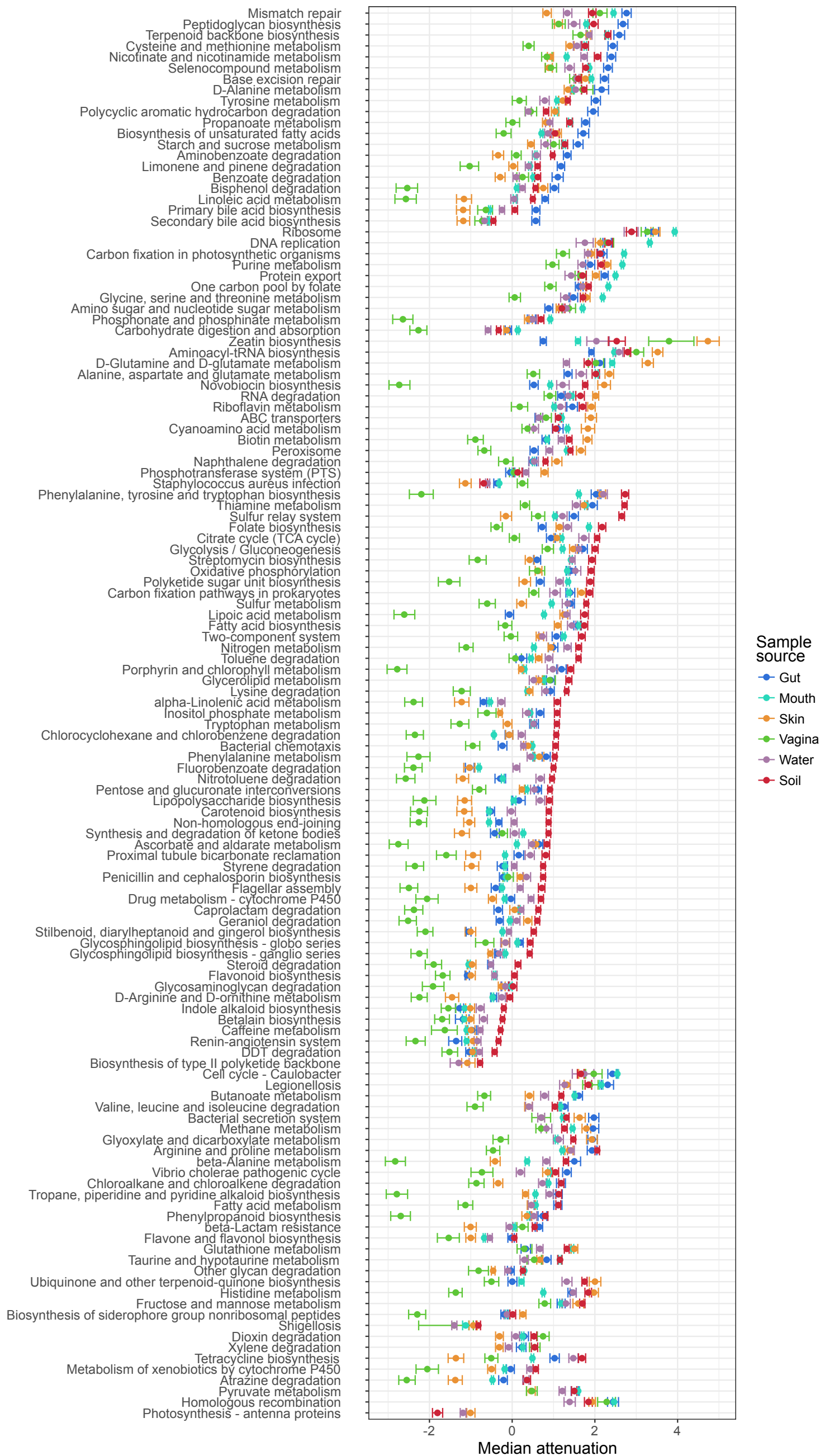

Supplement: Supplementary file 7 — Figure S4. Pathway-specific attenuation of all pathways by environment. (PDF 1657 kb) [file 40168_2018_425_MOESM7_ESM.pdf]

A

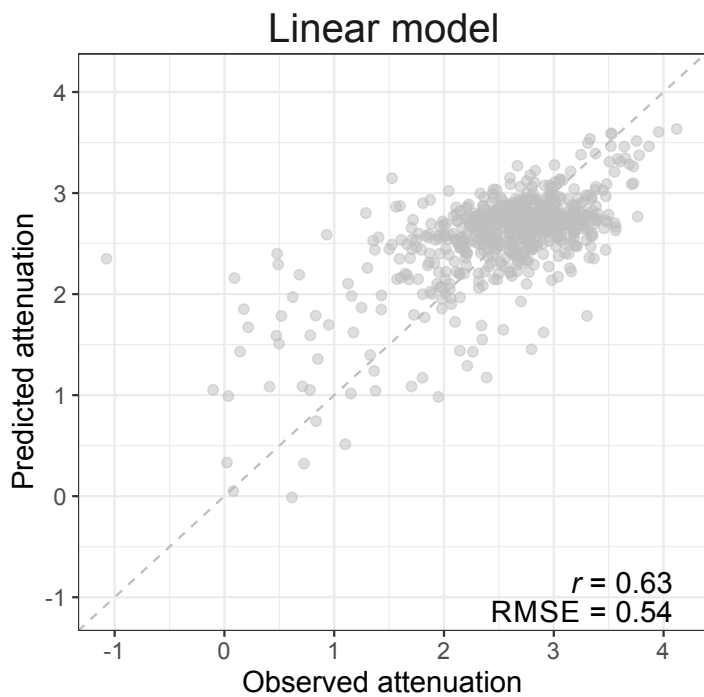

B

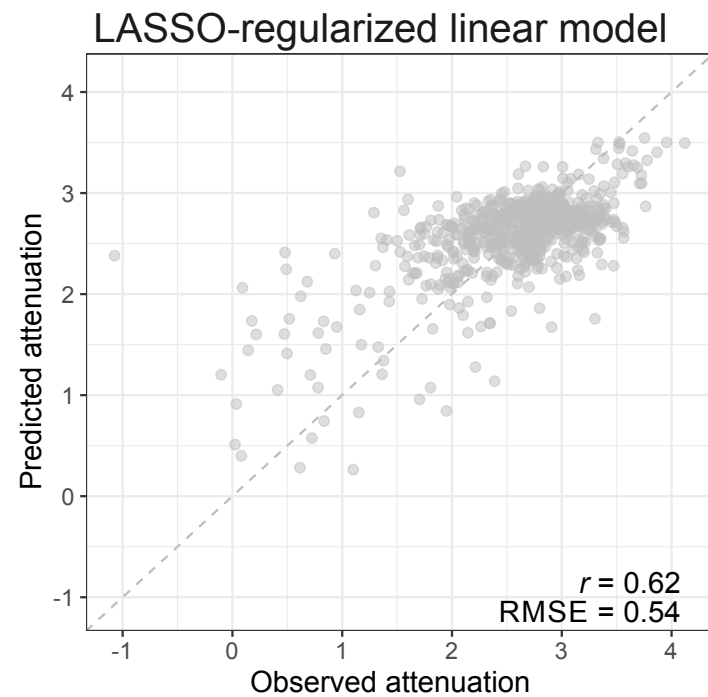

C

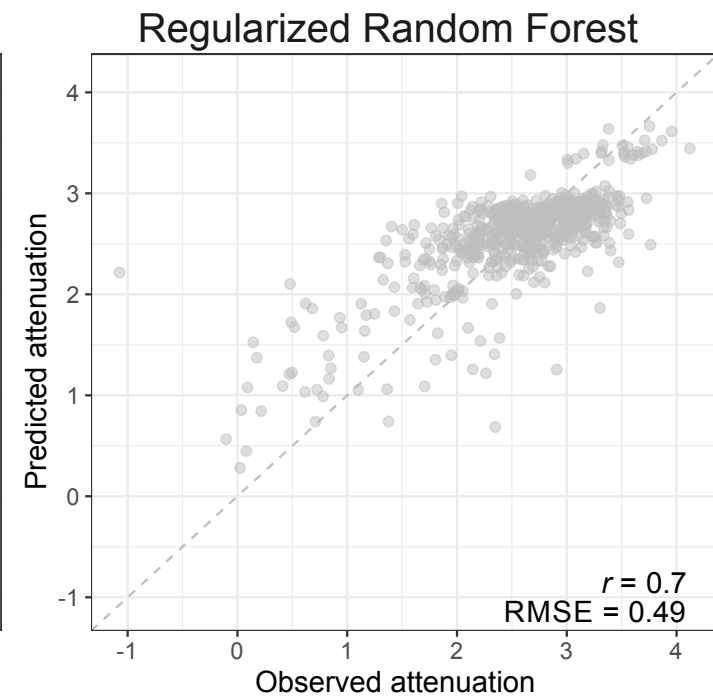

D

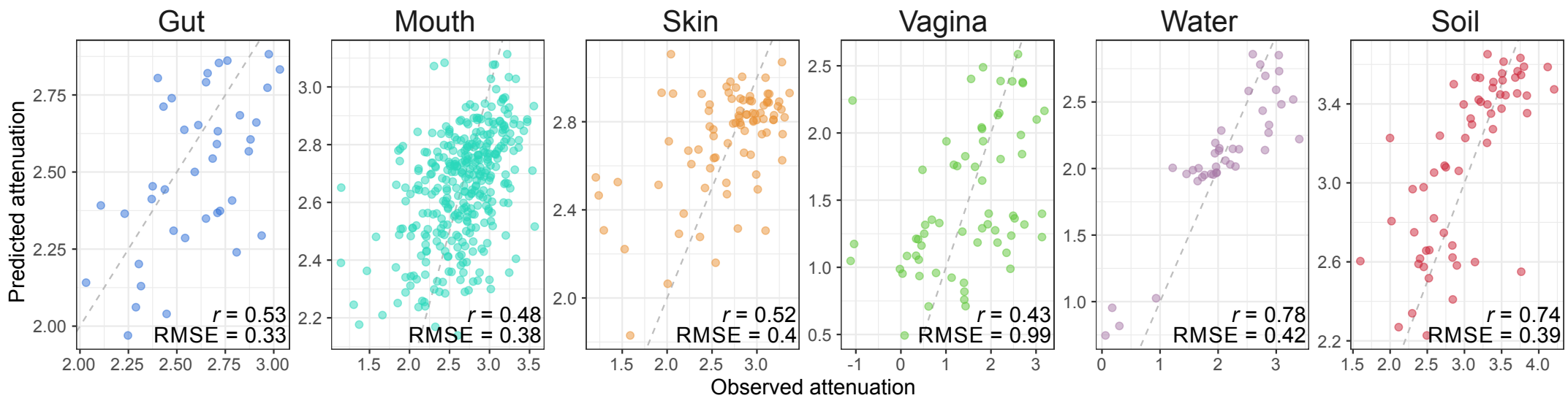

Supplement: Supplementary file 10 — Figure S6. Performance of GDF-based predictive models of attenuation, using an extended set of 45 GDFs. (PDF 1694 kb) [file 40168_2018_425_MOESM10_ESM.pdf]

A

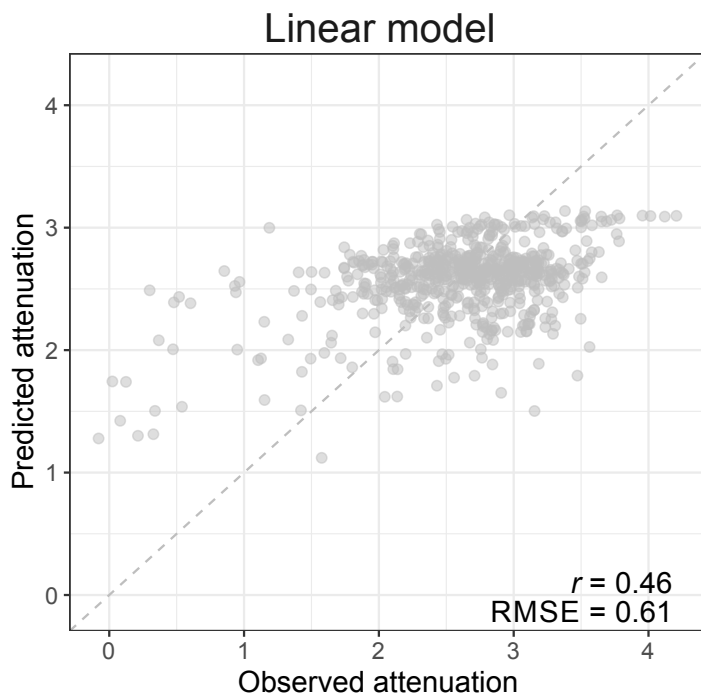

B

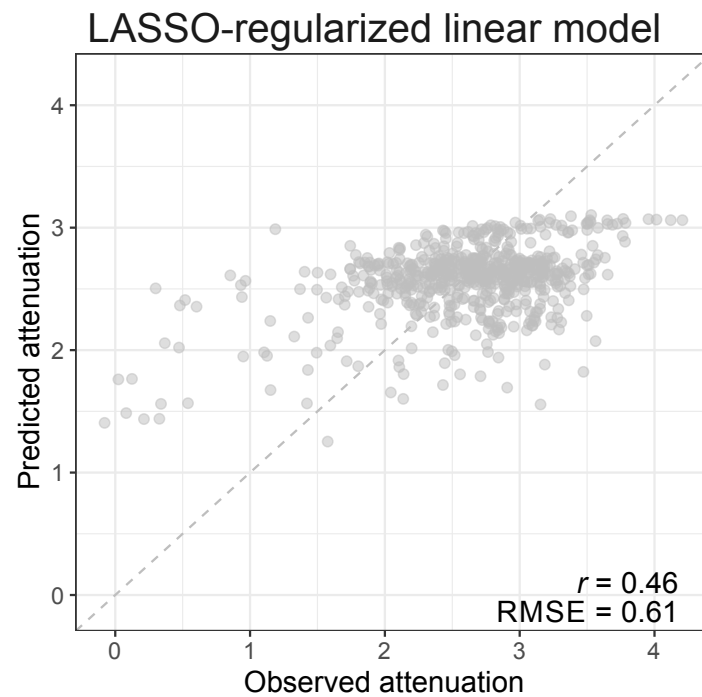

C

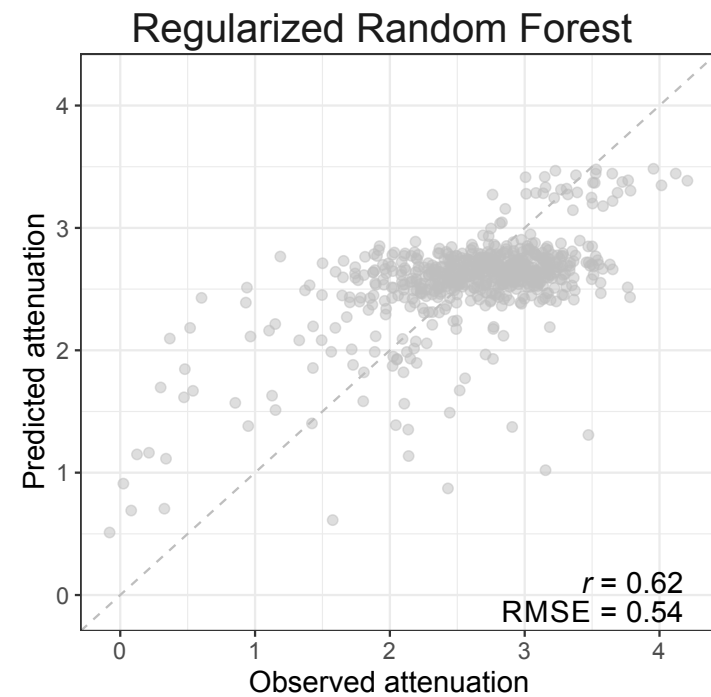

D

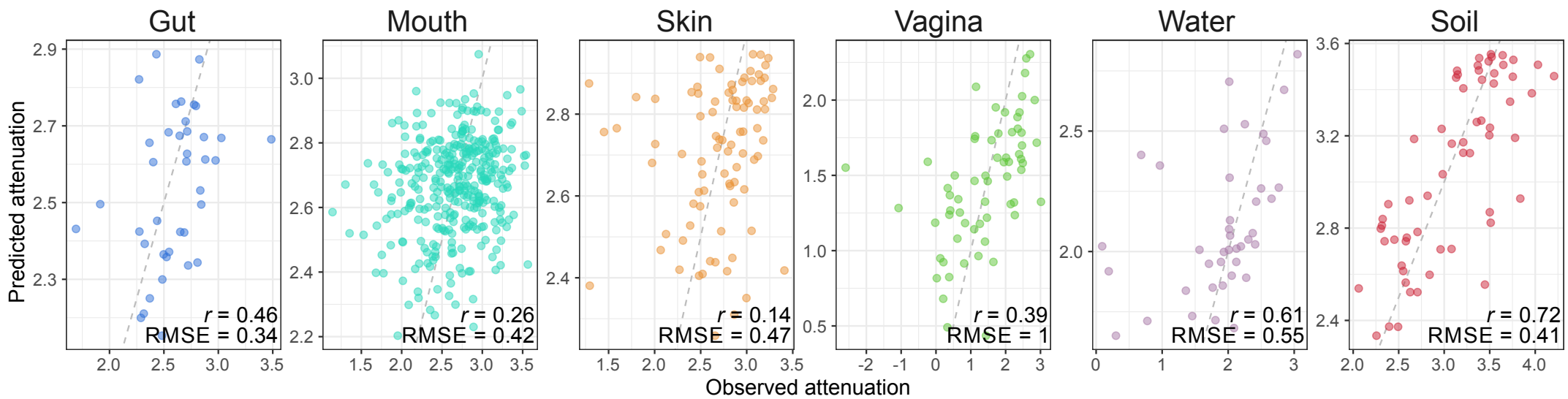

Supplement: Supplementary file 11 — Figure S7. Performance of GDF-based predictive models of attenuation with only 5 GDFs. (PDF 1677 kb) [file 40168_2018_425_MOESM11_ESM.pdf]
