# Supplementary material for: Taxa-function robustness in microbial communities
Source: Microbiome. 2018 Mar 2;6:45. doi: 10.1186/s40168-018-0425-4 (PMC5833107; doi:10.1186/s40168-018-0425-4)
Supplement: Supplementary file 8 — Figure S5. Correlations between gene distribution features. (PDF 45 kb) [file 40168_2018_425_MOESM8_ESM.pdf]

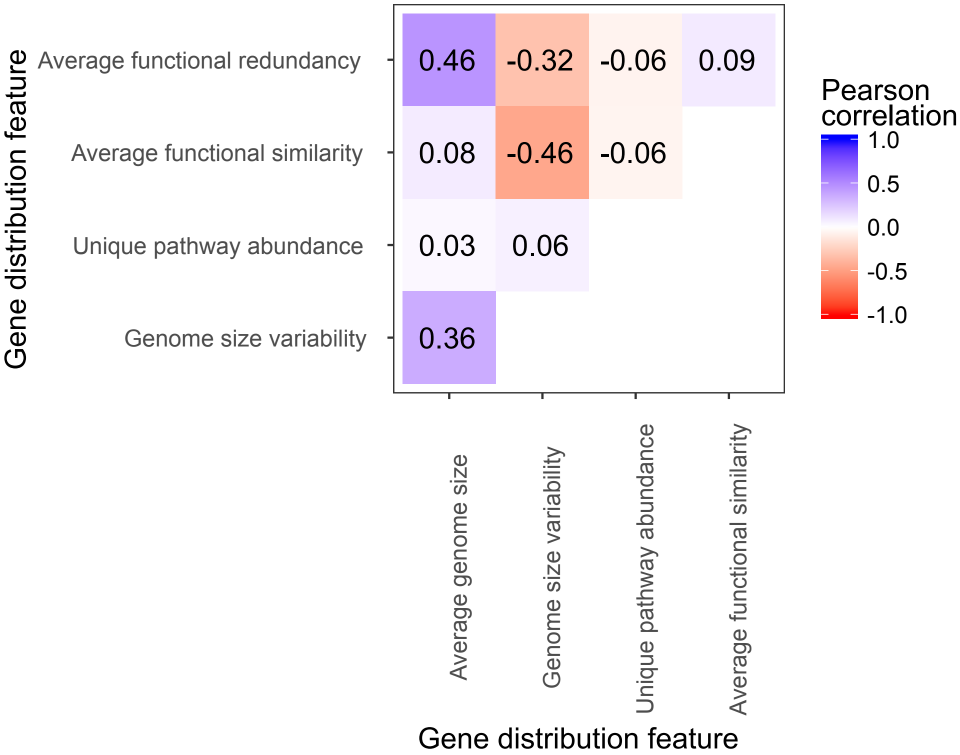

Average functional redundancy
Average functional similarity
Unique pathway abundance
Genome size variability
0.46
-0.32
-0.06
0.09
0.08
-0.46
-0.06
0.03
0.06
0.36
Average genome size
Genome size variability
Unique pathway abundance
Average functional similarity
Gene distribution feature
Gene distribution feature
Pearson correlation
1.0
0.5
0.0
-0.5
-1.0
